# Genome-wide comparisons reveal evidence for a species complex in the black-lip pearl oyster *Pinctada margaritifera* (Bivalvia: Pteriidae)

**DOI:** 10.1038/s41598-017-18602-5

**Published:** 2018-01-09

**Authors:** Monal M. Lal, Paul C. Southgate, Dean R. Jerry, Kyall R. Zenger

**Affiliations:** 1 Centre for Sustainable Tropical Fisheries and Aquaculture and College of Science and Engineering, James Cook University, Townsville Campus, Townsville, QLD 4811 Queensland Australia; 2 Australian Centre for Pacific Islands Research, Faculty of Science, Health, Education and Engineering, University of the Sunshine Coast, Maroochydore, QLD 4558 Queensland Australia; 3 School of Marine Studies, Faculty of Science, Technology and Environment, University of the South Pacific, Lower Laucala Campus, Laucala Bay Road, Suva, Fiji Islands

**Keywords:** Ecological genetics, Phylogenetics, Adaptive radiation, Taxonomy

## Abstract

Evolutionary relationships in the black-lip pearl oyster *Pinctada margaritifera* which is highly valued for pearl production remain poorly understood. This species possesses an 18,000 km Indo-Pacific natural distribution, and its current description includes six subspecies defined exclusively on morphological characters. To evaluate its taxonomic identity using molecular data, 14 populations in both the Indian and Pacific Oceans (n = 69), and the congeneric taxa *P*. *maxima* and *P*. *mazatlanica* (n = 29 and n = 10, respectively) were sampled. Phylogenomic reconstruction was carried out using both 8,308 genome-wide SNPs and 10,000 dominant loci (DArTseq PAVs). Reconstructions using neighbour-joining (Nei’s 1972 distances), maximum likelihood and Bayesian approaches all indicate that the taxonomy of *P*. *margaritifera* is quite complex, with distinct evolutionary significant units (ESUs) identified within Tanzanian and Iranian populations. Contrastingly, phylogenies generated for Pacific Ocean oysters resolved a large monophyletic clade, suggesting little support for two current morphological subspecies classifications. Furthermore, *P*. *mazatlanica* formed a basal clade closest to French Polynesian *P*. *margaritifera*, suggesting it may be conspecific. Collectively, these findings provide evidence that *P*. *margaritifera* comprises a species complex, perhaps as a result of population fragmentation and increased divergence at range limits.

## Introduction

**Figure 1:**
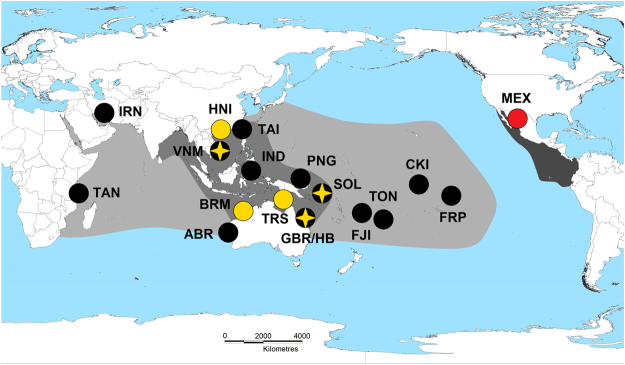
Natural distributions and sampling locations from where specimens of *P*. *margaritifera* (n = 69; solid black circles and black circles superimposed with yellow stars), *P*. *maxima* (n = 29; yellow circles and black circles superimposed with yellow stars) and *P*. *mazatlanica* (n = 10; solid red circle), were collected. The approximate known distributions of each species are presented in light grey (*P*. *margaritifera*), medium grey (*P*. *maxima*) and dark grey (*P*. *mazatlanica*) respectively; and adapted from Wada and Tëmkin^2^. Site codes represent the following locations; TAN: Mafia Island and Mtwara, Tanzania; IRN: Hendorabi Island, Iran; TAI: Checheng, Taiwan (*P*. *margaritifera*), HNI: Hainan Island, China (*P*. *maxima*); VNM: Nha Trang (*P*. *margaritifera*) and Phú Quốc (*P*. *maxima*), Vietnam; IND: Manado, Indonesia; ABR: Abrolhos Islands, Australia; BRM: Broome, Australia; TRS: Torres Strait, Australia; GBR/HB: Great Barrier Reef (*P*. *margaritifera*) and Hervey Bay (*P*. *maxima*), Australia; PNG: Kavieng, Papua New Guinea; SOL: Gizo Island, Solomon Islands; FJI: Kadavu, Savusavu, Lau and the Yasawa group, Fiji Islands; TON: Tongatapu, Tonga; CKI: Manihiki Atoll, Cook Islands; FRP: Arutua, French Polynesia and MEX: Guaymas, Mexico. This map was produced using ArcGIS release 10.2^76^.

The black-lip pearl oyster *Pinctada margaritifera* is a marine bivalve mollusc that has a broad Indo-Pacific distribution spanning ~18,000 km (Fig. 1), and is highly valued for cultured pearl and pearl shell production^1,2^. While several studies have examined contemporary population structure and connectivity in *P*. *margaritifera* ^3–13^, none have investigated its range-wide evolutionary history and species identity using molecular data. An understanding of the taxomomic status and genetic structure of this bivalve is important for management of the species given its community and economic value, but also more broadly to elucidate the drivers of evolutionary relationships between widely separated populations of an extensively-distributed marine invertebrate^14–16^.

Species-level taxonomic relationships in the genus *Pinctada* had remained unresolved, particularly because many earlier descriptions either heavily, or exclusively relied on morphological descriptions of shells, which are now known to display considerable phenotypic, developmental and environmental plasticity^2,17,18^. With the increasing use of molecular tools to resolve both higher and lower level relationships in this taxon, clarity in the taxonomic identity of several species important for cultured pearl production is being established. An example of this which is still pending ultimate resolution, is the status of the *P*. *fucata*/*martensii*/*radiata*/*imbricata* (Akoya pearl oyster) species complex. It is currently recognised that this group may comprise one cosmopolitan, circum-globally distributed species, possessing a very high degree of intraspecific variation across its range^1,2^.

Given the phenotypic and adaptive plasticity, as well as morphological diversity present in Pteriid pearl oysters^17–19^, and that shell shape and size differences are apparent between populations of *P*. *margaritifera* ^2,17,20^, molecular data are required to elucidate Evolutionary Significant Units (ESUs)^21^ in this species. For the purpose of this study, we define ESUs after Funk *et al*.^21^ and Crandall *et al*.^22^, as being a classification of populations possessing substantial reproductive isolation and/or adaptive differences, so that the population represents a significant evolutionary component of the species. Due to the degree of molecular intraspecific variation documented in *P*. *margaritifera*, localised studies have suggested that it might constitute a species complex^2,20,23,24^. The term species complex is used to describe populations where a group of organisms may represent more than one species, and/or where species boundaries cannot be discerned with certainty^25–27^. In the case of *P*. *margaritifera*, the disagreement between current molecular^14^ and morphological^2,17,20^ information could indicate the presence of a species complex involving several discrete ESUs, which require identification and delimitation.

Particular studies which point to a *P*. *margaritifera* species complex include a thorough morphological and molecular characterisation of the superfamily Pterioidea by Tëmkin^23^, who reported that *P*. *mazatlanica* formed an unresolved clade with *P*. *margaritifera*, suggesting their conspecificity. In the Persian Gulf, Ranjbar, *et al*.^20^ using mitochondrial COI data discovered that *P*. *m*. var. *persica* formed a divergent ESU, and suggested its reclassification as *P*. *persica*, while *P*. *m*. var. *zanzibarensis* from Mauritius formed a basal clade to French Polynesian and Japanese specimens^24^.

Descriptions of *P*. *margaritifera* include a total of six subspecies^16^, along with a former seventh (*P*. *margaritifera mazatlanica*), that has since been elevated as a distinct species; *P*. *mazatlanica* (Hanley, 1856)^2,24,28,29^. The six subspecies of *P*. *margaritifera* are described exclusively on the basis of morphological characters^16,28^, and are closely associated with their geographic type locations (see Table 1 for a summary). In the Pacific basin, Hawaiian populations are known as *P*. *margaritifera* var. *galstoffi*, Cook Islands and French Polynesian individuals *P*. *m*. var. *cummingi*, and all Central and Western Pacific specimens *P*. *m*. var. *typica*. Indian Ocean populations are represented by *P*. *m*. var. *persica* (Persian Gulf), *P*. *m*. var. *erythraensis* (Red Sea) and *P*. *m*. var. *zanzibarensis* (East Africa, Madagascar and Seychelle Islands). Recent efforts to characterise distribution-wide population genetic structure in *P*. *margaritifera* ^14^ indicate a high degree of homogeneity within the Pacific basin, bringing into question the current subspecies classifications of *P*. *m*. var. *typica* and *P*. *m*. var. *cummingi*. Conversely, populations examined from the Indian Ocean displayed substantial vicariance from Pacific Ocean demes, possibly supporting the existence of distinct ESUs in that region.

**Table 1:** Described subspecies of the black-lip pearl oyster as summarised by Gervis and Sims^16^.

Subspecies	Authority	Regional distribution	References	Remarks
*P*. *margaritifera* var. *cummingi*	(Reeve, 1857)	Cook Islands and French Polynesia	Coeroli, *et al*.^77^ Galstoff^78^ Gug^79^ Hedley^80^ Jameson^28^ Ranson^34^ Saville-Kent^81^	Classification appears to be based on morphology, most recently described by Jameson^28^ and Hynd^82^.
*P*. *margaritifera* var. *typica*	(Linnaeus, 1758)	Ryukus Is., Japan; Taiwan, Australia (GBR), Fiji Is.	Hedley^80^ Hynd^82^ Saville-Kent^81^	Classification appears to be based on morphology, most recently described by Jameson^28^ and Hynd^82^.
*P*. *margaritifera* var. *galstoffi*	(Bartsch, 1931)	Hawai’i	Cahn^83^ Galstoff^78^ Wada and Tëmkin^2^	Classification appears to be based on morphology. Originally described by Bartsch^33^ as *P*. *galstoffi*.
*P*. *margaritifera* var. *erythraensis*	(Jameson, 1901)	Red Sea	Jameson^28^	Classification appears to be based on morphology, most recently described by Jameson^28^. Length/weight relationships characterised by Elamin and Elamin^84^.
*P*. *margaritifera* var. *zanzibarensis*	(Jameson, 1901)	East Africa, Madagascar, Seychelle Is.	Jameson^28^	Classification appears to be based on morphology, most recently described by Jameson^28^.
*P*. *margaritifera* var. *persica*	(Jameson, 1901)	Persian Gulf	Jameson^28^	Ranjbar, *et al*.^20^ suggest that this subspecies is an independent ESU, and should be revised as a separate species named *P*. *persica*.
*P*. *margaritifera* var. *mazatlanica*	(Hanley, 1856)	Baja California, Panama Bay	Jameson^28^ Hanley and Wood^29^	Currently recognised as a species in its own right as *P*. *mazatlanica*, however, the most recent phylogenetic reconstruction by Tëmkin^23^ suggests conspecificity with *P*. *margaritifera*.

The goals of the present study were to use genome-wide SNP and dominant marker data to resolve species-level phylogenetic relationships and the presence of ESUs within *P*. *margaritifera*, and provide information on its taxonomic identity. This investigation is the first of its kind to assess individuals spanning the distributional range of the black-lip pearl oyster, and the data generated has high utility for informing regional spatial marine management strategies for conservation and aquaculture efforts.

## Results

Population and species-level relationships were reconstructed using neighbour-joining (NJ), maximum-likelihood (ML) and Bayesian inference approaches. Additionally, Nei’s (1973) minimum (*D*_m_) and unbiased (*D*, 1972) genetic distances were computed for the SNP and dominant marker (DArTseq PAV) datasets respectively (see methods).

### Genotyping and SNP/PAV filtering

The raw SNP dataset contained a total of 23,599 SNPs genotyped across all 118 individuals, at call rates ranging from 20–100%. The first filtering step undertaken to remove duplicate SNPs within the same sequence tag resulted in the removal of 5,634 SNPs (24% loss), after which the dataset was filtered for call rate (>70%), average PIC (>1%), MAF (>2%) and average repeatability (>95%). A total of 2 loci were monomorphic within a single taxon, and subsequently removed. These steps collectively resulted in the retention of 8,308 SNPs, across 107 individuals. The raw PAV dataset contained a total of 42,159 variant scores across all individuals, at call rates ranging from 90–100%. No duplicate genomic loci were represented in the dataset, and following filtering for call rate (100%), MAF (>10%) and average reproducibility (>98%), 12,212 PAVs remained. No individuals were lost due to poor call rates as with the SNP loci, however the dataset was trimmed to 10,000 PAVs to maximise computational efficiency. All 118 taxa were included in the analyses, and *Pt*. *penguin* retained as the outgroup taxon.

### Phylogenomic reconstruction

#### NJ and ML approaches

**Figure 2:**
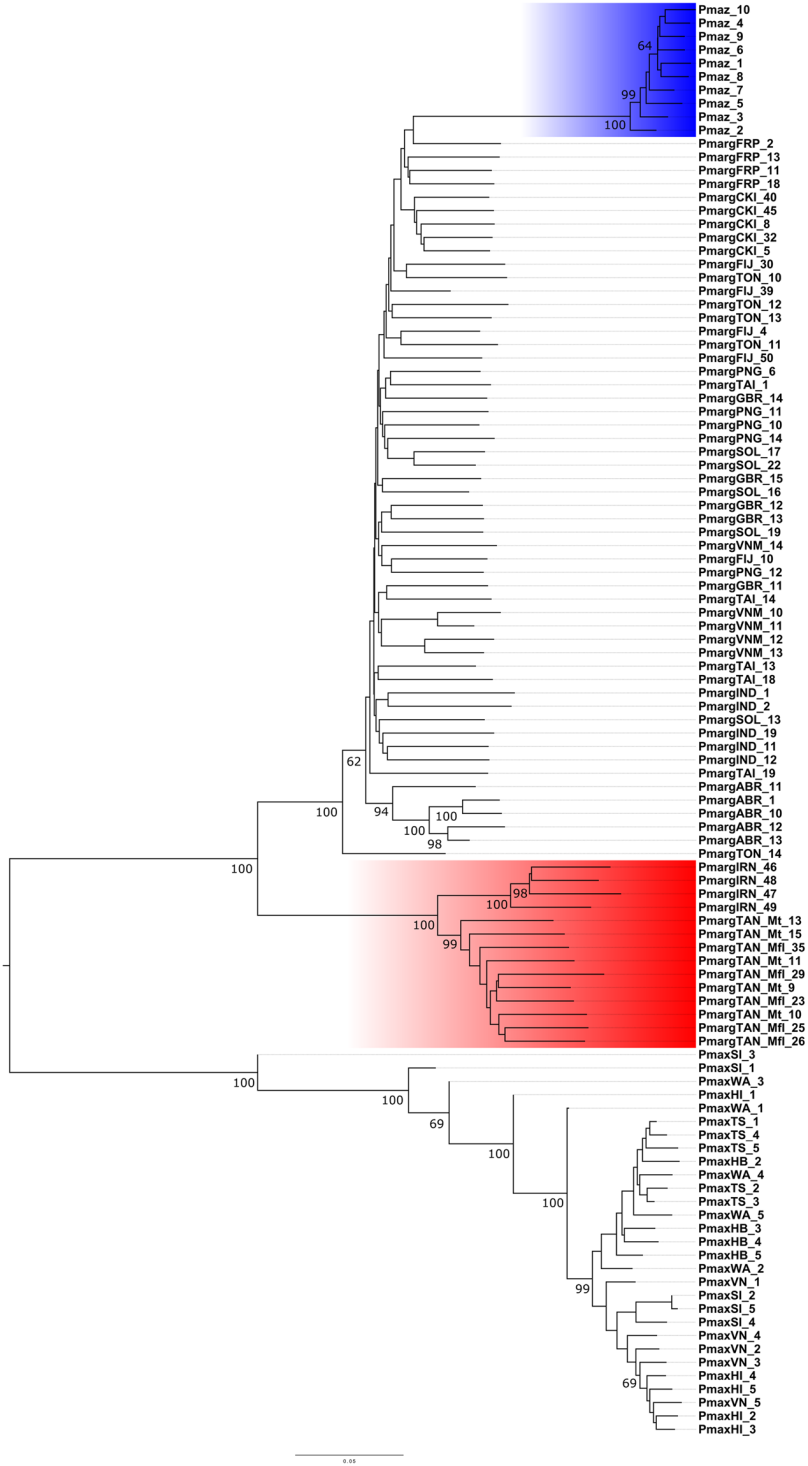
Neighbourjoining tree generated in MEGA6^60^ using 8,308 SNPs based on Nei's 1972 genetic distances. *P*. *maxima* was used as the outgroup taxon. Values reported at nodes indicate bootstrap support at a threshold higher than 60 (1,000 bootstraps used). Clades for *P*. *mazatlanica* and Iranian with Tanzanian *P*. *margaritifera* are highlighted in blue and red respectively.

**Figure 3:**
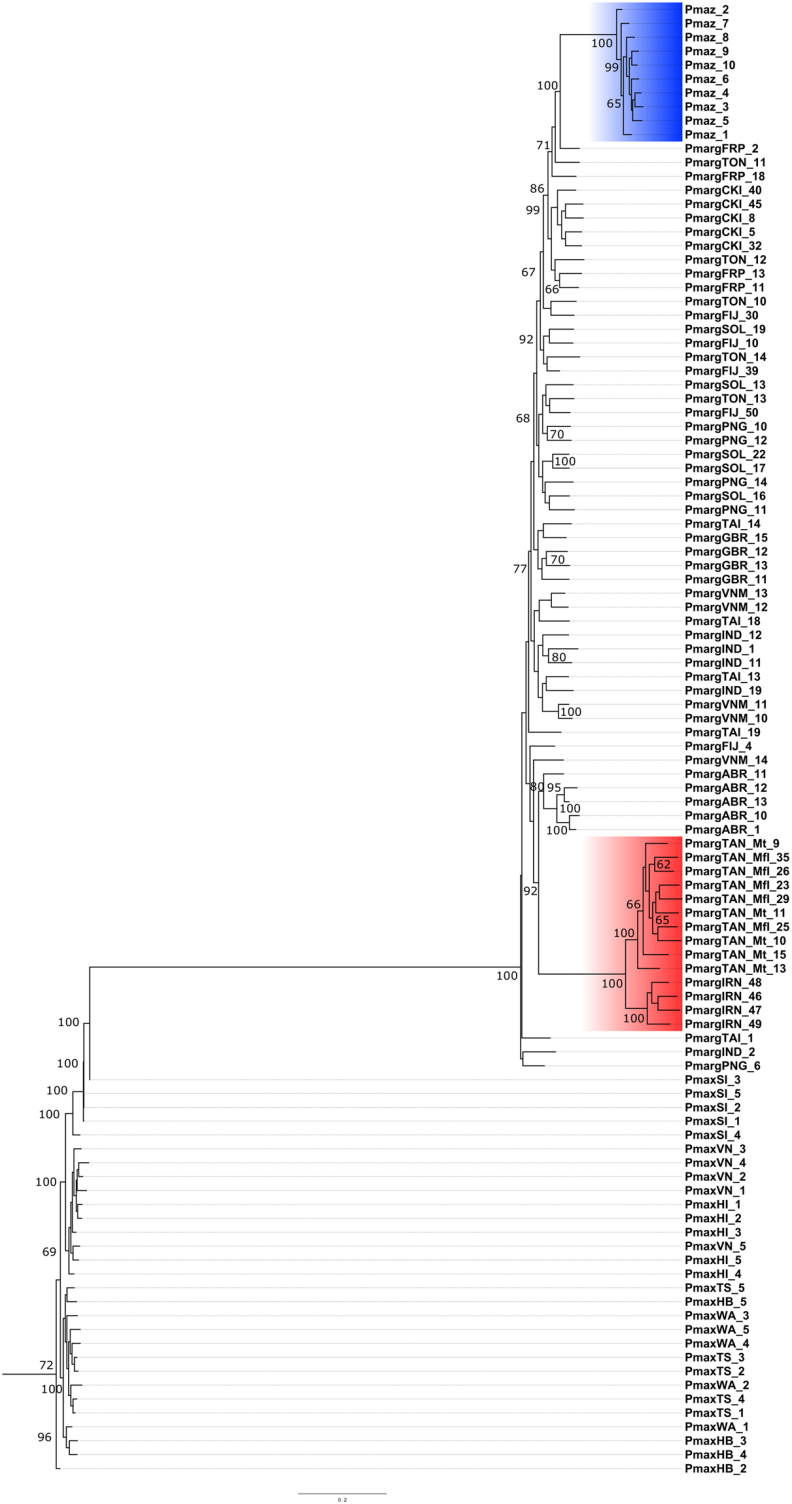
Maximumlikelihood tree generated using the SNPhylo package^61^, with 100,000 bootstraps and 8,308 SNPs. *P*. *maxima* was used as the outgroup taxon. Values reported at nodes indicate bootstrap support at a threshold higher than 65. Clades for *P*. *mazatlanica* and Iranian with Tanzanian *P*. *margaritifera* are highlighted in blue and red respectively.

Both NJ and ML reconstructions (Figs 2 and 3) produced nearly identical topologies, demonstrating clear separation of *P*. *margaritifera* and *P*. *maxima* into species-level clades. Interestingly, within the larger monophyletic *P*. *margaritifera* clade, all individuals of *P*. *mazatlanica* formed a single basal group with short internal branch lengths, and node support for this division in both NJ and ML reconstructions was high (100). Similarly, all Tanzanian with Iranian *P*. *margaritifera* also formed a distinct monophyletic group, which was basal to the larger monophyletic clade resolved for all other *P*. *margaritifera* samples. As with the *P*. *mazatlanica* clade, high bootstrap node support (100) and short internal branch lengths were resolved for this division. Overall, the shallow levels of divergence documented among Pacific Ocean *P*. *margaritifera* populations suggests their conspecificity, and consequently does not support the current subspecies classifications of *P*. *margaritifera* var. *typica* and *P*. *m*. var. *cummingii*. Monophyly of *P*. *mazatlanica*, as well as of the combined Iranian and Tanzanian populations of *P*. *margaritifera* within the larger *P*. *margaritifera* cluster, is suggestive of discrete ESUs present in these locations. Examination of Iranian and Tanzanian specimens in isolation indicates a paraphyletic relationship between the two groups, and underscores the need for further work to resolve evolutionary relationships among Indian Ocean black-lip pearl oyster populations.

#### Bayesian approach for PAV dataset

**Figure 4:**
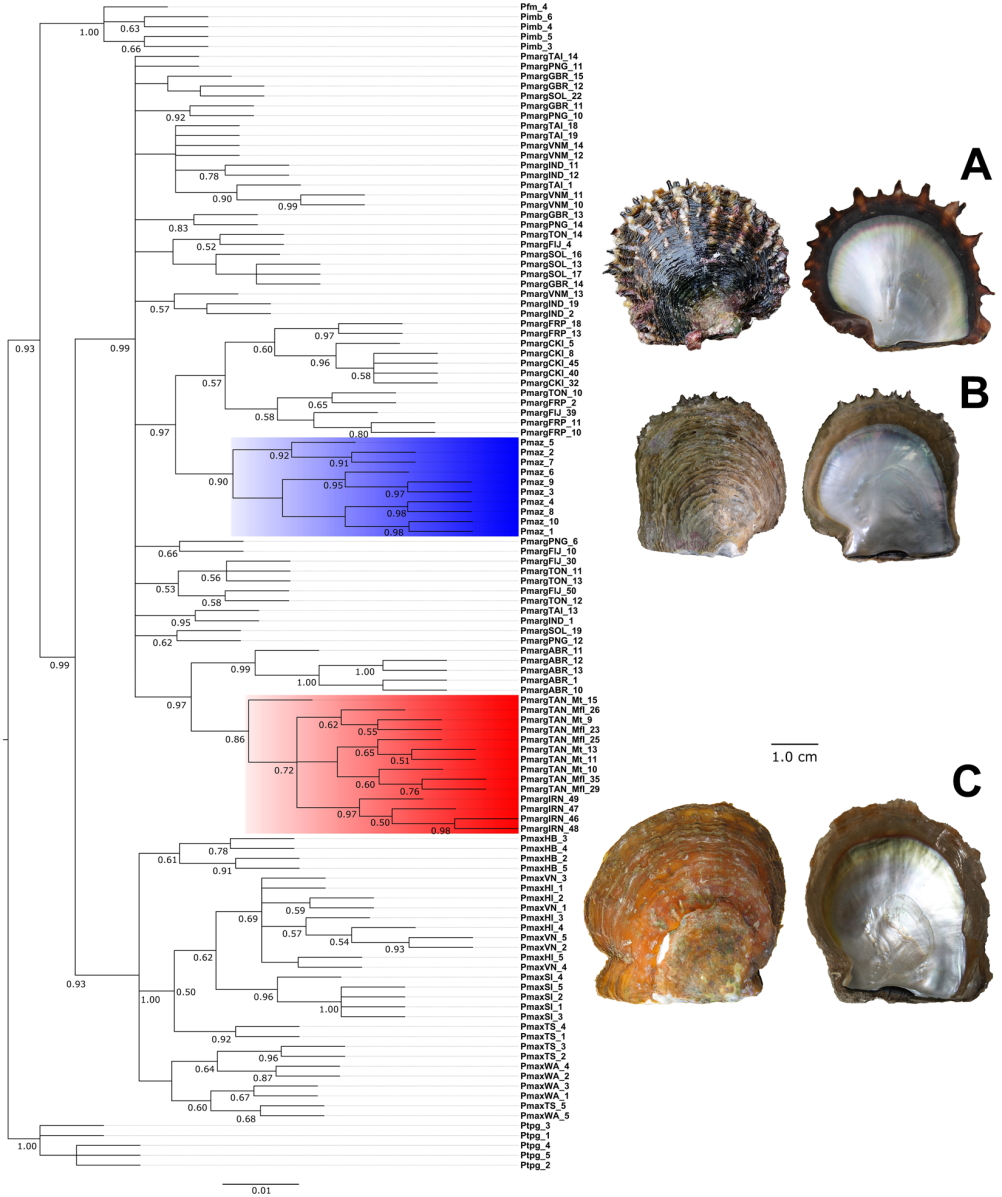
Bayesian reconstruction generated using 10,000 DArTseq PAVs in MrBayes v.3.2^69^. The consensus tree reported here was generated from 1,141 of the most credible set of trees (p = 0.01), using a 50% majority consensus rule. Posterior probabilities ≥0.50 for node support are indicated below the branches. Clades for *P*. *mazatlanica* and Iranian with Tanzanian *P*. *margaritifera* are highlighted in blue and red respectively. Representative specimens of *P*. *margaritifera* (**A;** Savusavu, Fiji Is.), *P*. *mazatlanica* (**B;** Guaymas, Mexico) and *P*. *maxima* (**C;** Bali, Indonesia), are shown to illustrate gross differences in shell morphology.

A total of 480,002 trees were sampled from both runs, and following discard of the burn-in set, 414,101 credible trees remained for calculation of posterior probabilities. The final average standard deviation of split frequencies achieved was 0.013, with an average potential scale reduction factor (PSRF) for parameter values of 1.000. Within the final set of credible trees, cut-off thresholds of individual (p = 0.000) and cumulative (P < 0.009) posterior probabilities were implemented, to select 1,141 highly credible trees with which to construct a consensus phylogram (Fig. 4). The reconstruction resolved three major groups, corresponding to established species-level divisions of *P*. *fucata martensii* with *P*. *imbricata* (Akoya species complex), *P*. *margaritifera* and *P*. *maxima*. As with the NJ and ML reconstructions, two distinct monophyletic groups were resolved within the larger *P*. *margaritifera* clade. However, for the sub-group which included *P*. *mazatlanica*; French Polynesian, Cook Islands, Fijian and Tongan *P*. *margaritifera* were found to nest together. Similarly, all Abrolhos Islands individuals clustered alongside the Tanzanian and Iranian individuals. It is possible that due to the lower discriminating power of the PAV dataset compared to SNPs at the species level, these more distal relationships were not able to be resolved. This may also explain the similar branch tip lengths observed for all taxa in the final phylogram.

### Assessment of genetic distances between population groups and species

Genetic distance estimates differed between the SNP and PAV datasets due to the different marker systems (Table 2), although overall trends for the majority of pairwise comparisons were similar. Nei’s (1973) minimum genetic distances (*D*_m_) values obtained from the SNP dataset indicated that *P*. *maxima* from all locations were significantly and substantially divergent from all *P*. *margaritifera* and *P*. *mazatlanica* populations, (*D*_m_ > 0.22; p < 0.05). Among sampling sites for *P*. *maxima*, estimates ranged from 0.023 (Hervey Bay and Vietnam) to 0.069 (Hervey Bay and Solomon Islands), confirming their conspecificity. Pacific Ocean populations of *P*. *margaritifera* were largely homogenous (*D*_m_ = 0.006–0.038; p < 0.05), and only showed signs of divergence when compared against Indian Ocean populations from Tanzania (*D*_m_ = 0.076–0.089) and Iran (*D*_m_ = 0.069–0.113). Interestingly, all *P*. *mazatlanica* estimates (*D*_m_ = 0.057–0.133; p < 0.05), fell within the range limits of all between-site *P*. *margaritifera* estimates (*D*_m_ = 0.006–0.113; p < 0.05).

**Table 2:** Pairwise Nei’s minimum (*D*_m_, 1973), and Nei’s unbiased (*D*, 1972) genetic distance estimates between sampling locations and species, presented below and above the diagonal for SNP and DArTseq PAV datasets respectively.

	SITE	*P*. *margaritifera*								*P*. *maxima*						*P*. *mazatlanica*
		ABR	CKI	FRP	FIJ + TON	PNG + SOL + GBR	TAI + VNM + IND	TAN	IRN	HB	HI	SI	TS	VNM	WA	MEX
*P*. *margaritifera*	ABR	—	0.017	0.013	0.013	0.012	0.011	0.035	0.022	0.157	0.173	0.279	0.171	0.141	0.177	0.051
	CKI	0.038	—	0.002	0.002	0.003	0.005	0.036	0.018	0.142	0.158	0.262	0.156	0.126	0.161	0.037
	FRP	0.036	0.027	—	0.000	0.001	0.002	0.033	0.014	0.136	0.152	0.255	0.149	0.120	0.155	0.034
	FIJ + TON	0.024	0.018	0.018	—	0.000	0.001	0.031	0.013	0.138	0.154	0.258	0.152	0.122	0.158	0.035
	PNG + SOL + GBR	0.024	0.018	0.017	0.007	—	0.000	0.029	0.012	0.137	0.153	0.257	0.151	0.122	0.157	0.037
	TAI + VNM + IND	0.022	0.020	0.018	0.009	0.006	—	0.026	0.011	0.138	0.154	0.257	0.151	0.122	0.157	0.038
	TAN	0.082	0.089	0.089	0.081	0.078	0.076	—	0.013	0.172	0.189	0.296	0.186	0.156	0.193	0.068
	IRN	0.103	0.113	0.111	0.104	0.101	0.099	0.069	-	0.141	0.157	0.261	0.155	0.126	0.161	0.047
*P*. *maxima*	HB	0.269	0.286	0.287	0.278	0.277	0.277	0.263	0.255	—	0.023	0.080	0.006	0.023	0.008	0.182
	HI	0.259	0.276	0.277	0.267	0.266	0.266	0.256	0.248	0.032	—	0.066	0.029	0.005	0.037	0.199
	SI	0.232	0.248	0.247	0.238	0.238	0.237	0.235	0.229	0.069	0.064	—	0.085	0.078	0.093	0.307
	TS	0.265	0.282	0.283	0.273	0.273	0.273	0.262	0.255	0.025	0.037	0.068	—	0.032	0.009	0.197
	VN	0.271	0.288	0.289	0.279	0.279	0.278	0.265	0.257	0.023	0.029	0.064	0.028	—	0.039	0.165
	WA	0.256	0.273	0.274	0.264	0.265	0.264	0.254	0.246	0.032	0.042	0.067	0.037	0.037	—	0.203
*P*. *mazatlanica*	MEX	0.072	0.063	0.058	0.059	0.057	0.058	0.114	0.133	0.289	0.278	0.252	0.284	0.289	0.274	—

*D*_m_ estimates were computed using Genetix v.4.05.2^56^, while *D* estimates were computed in AFLP-SURV v.1.0^58^. All values are significant (p < 0.05) following permutation. Population groups for *P*. *margaritifera* were assigned according to Lal, *et al*.^14^, while groups for *P*. *maxima* were retained according to discrete sampling locations. Sampling site codes are identical to those detailed for Fig. 1, except for the following *P*. *maxima* sites: HI = Hainan Is., China; SI = Solomon Is.; VN = Vietnam; WA = Broome, Western Australia.

Nei’s (1972) unbiased genetic distance (*D*) estimates also indicated clear separation of *P*. *maxima* from *P*. *margaritifera* (*D* = 0.126–0.296; p < 0.05), and *P*. *mazatlanica* (*D* = 0.165–0.307; p < 0.05) samples, in keeping with the trend observed for *D*_m_ values. Similarly, broad-scale divergence between *P*. *mazatlanica* and *P*. *margaritifera* was not apparent, although the Abrolhos, Tanzanian and Iranian populations did display slightly increased values (0.051, 0.068 and 0.047, respectively), compared to the range estimated for Pacific populations (*D* = 0.034–0.038, p < 0.05), reflecting divergence between ocean basins.

## Discussion

The current study represents the most comprehensive evaluation of evolutionary relationships in the black-lip pearl oyster, incorporating two independent high density genome-wide marker sets and sample representation from the entire species distribution. It is clear that the taxonomy of *P*. *margaritifera* is more complex than previously thought, suggesting that its status may require re-examination.

The presence of morphological differences^17,28^ between locations in *P*. *margaritifera* is unsurprising, considering that the species distribution spans over 18,000 km across heterogeneous habitats^2,14^, and that bivalve molluscs can display very high levels of phenotypic plasticity^24,30^. The high degree of morphological variation and overlapping geographical distributions of many bivalves due to high larval dispersal capability, however, can make the delimitation of population and species boundaries problematic, highlighting the need for molecular information to resolve these differences. The data presented in the current study indicates varying degrees of support for the current morphological subspecies classifications for *P*. *margaritifera*.

In the Pacific Ocean, the existence of the morphological subspecies *P*. *m*. var. *typica* and *P*. *m*. var. *cummingii* is questionable. Previous work^14^ to examine genetic structure in the Pacific basin supports this finding, as results showed very high gene flow between *P*. *margaritifera* populations separated by several thousands of kilometres. While five discrete Pacific Ocean stocks were identified in that study, populations remained sufficiently undifferentiated in the current study to resolve any subspecies groupings. It is possible that the morphological subspecies recorded in the Pacific^17,28^ may be the result of local adaptation and habitat differences. Lal *et al*.^14^ detected signatures of selection between all five stocks of *P*. *margaritifera* identified in the Pacific, and given the adaptive capacity of many bivalve taxa^31,32^, it is possible that local selective pressures may give rise to the morphological variability observed. This information was used as the basis for recommending separate regional fishery management of these populations, applying criteria established by Funk *et al*.^21^ and Crandall *et al*.^14,21,22^.

Despite considerable effort, it was not possible to obtain specimens of Hawaiian *P*. *margaritifera* for inclusion in the current study, and therefore examination of the status of the third and last Pacific Ocean subspecies described in the literature; *P*. *m*. var. *galstoffi* ^2,33^, remained out of reach. However, given the degree of genetic homogeneity between other Pacific populations across similar spatial scales, it is possible that Hawaiian populations could also display shallow levels of divergence, with the strength of migration between these locations determining their degree of isolation. A future study incorporating these populations will be able to address this question.

The discovery that *P*. *mazatlanica* specimens comprised a basal group nested within *P*. *margaritifera* is interesting, given the lack of an overlap in known species range limits^2^. In the light of the substantial trans-Pacific dispersal ability of this species as discussed earlier, it is entirely possible that recruits originating from eastern Pacific populations could find their way to the Gulf of California, thus maintaining gene flow. Early descriptions of *P*. *mazatlanica* using morphological characters had in fact classified it as a subspecies of *P*. *margaritifera* (*P*. *margaritifera mazatlanica*; and it was noted that its shell morphology appears to be an intermediate form between *P*. *margaritifera* and *P*. *maxima* ^2,28,29,34^).

Assessment of the population genetic structure of *P*. *mazatlanica* still remains unresolved. Arnaud, *et al*.^35^ discovered that mtDNA nucleotide divergence (COI and 12s rRNA) between locations ranged from 0.12–1.3%, while divergence from three individuals of *P*. *margaritifera* reached ~4%. Importantly, divergence at mtDNA loci across the broader distribution of *P*. *margaritifera* remains unknown. Subsequent examinations of species-level taxonomy within the genus *Pinctada* ^23^ using multiple nuclear and mitochondrial loci, found that *P*. *mazatlanica* formed an unresolved clade with *P*. *margaritifera*, in concordance with a later reconstruction by Cunha, *et al*.^24^, although in their study the latter authors state that results supported the present species-level classification. The genome-wide data presented here provides further strong evidence that these taxa might be conspecific. These results also support a further observation by Cunha, *et al*.^24^, which suggests that French Polynesian *P*. *margaritifera* are more closely related to *P*. *mazatlanica* than Indian Ocean specimens. This pattern of differentiation in the data also extended to populations located in the western Pacific in the data, indicating that a clinal effect related to geographic separation may be present, as seen in *Crassostrea* spp. oysters^30^.

The lower than expected levels of divergence separating *P*. *mazatlanica* from *P*. *margaritifera*, despite the morphological differences between the two taxa, raises several questions as to the processes driving their divergence, or maintaining genetic structure. One possibility is that *P*. *mazatlanica* is a distinct ESU within *P*. *margaritifera* (see criteria outlined by Funk *et al*.^21^ for evaluating ESUs), and that its morphological differences are a consequence of phenotypic and adaptive plasticity. Another scenario is that *P*. *mazatlanica* may be undergoing incipient speciation^36^, which has been documented in other broadcast spawning marine invertebrates, including bivalves^37,38^. To resolve these questions, a fine-scale study incorporating range-wide samples of *P*. *mazatlanica*, together with specimens from marginal eastern Pacific *P*. *margaritifera* populations (e.g. Hawaii, Kiribati and French Polynesia), is required.

All three phylogenetic reconstruction methods applied in the current study resolved a pattern of paraphyly for Iranian and Tanzanian specimens respectively, which corresponds with the morphological subspecies descriptions of *P*. *m*. var. *zanzibarensis* (Zanzibar, Madagascar and eastern African coastline) and *P*. *m*. var. *persica* (Persian Gulf only)^28^. This difference in evolutionary trajectories from Pacific Ocean specimens also explains the divergence observed in examination of range-wide population genetic structure in *P*. *margaritifera* ^14^. Separation of Persian Gulf populations as a distinct ESU was also detected by Ranjbar, *et al*.^20^, who suggested their reclassification as a species in its own right named *P*. *persica*. While the findings here do not indicate that specimens from Iran were sufficiently divergent to warrant this elevation in taxonomic rank (see criteria outlined by Crandall *et al*.^22^), further research is required to ascertain the degree of isolation of Persian Gulf populations from specimens living in the Red Sea (*i*.*e*. *P*. *m*. *erythraensis*), as well as the broader Indian Ocean.

It is clear that dense sampling of the Indian Ocean is required, as the present study was only able to assess three marginal populations sampled at its geographic limits. At the eastern extent of the Indian Ocean, specimens collected from the Abrolhos Islands (Western Australia) formed a weakly monophyletic clade, which nested closest to the Tanzanian and Iranian specimens, suggesting a restriction in gene flow between the Indian and Pacific Oceans^14^. This observation may also indicate the presence of an Indian-Pacific Ocean genetic break, however a future study incorporating a range of samples from both the Indian Ocean basin centre and periphery is required for confirmation. Additionally, Lal *et al*.^14^ recommended separate fishery management given the levels of divergence observed between these Indian Ocean populations, and the findings presented here add weight to the hypothesis that they have evolved as separate lineages. The criteria outlined by Crandall *et al*.^22^ are particularly useful for application of fishery management action for *P*. *margaritifera*, as they permit the preservation of adaptive diversity and evolutionary processes across the species’ range.

The possibility that *P*. *margaritifera* may constitute a species complex has been suggested by a number of studies, where distinct ESUs have been discovered during localised investigations of genetic structure^2,20,24^. The current study incorporating samples spanning the extent of the species distribution contributes further evidence to support this taxonomic classification, and highlights the need for further research to investigate segments of the species range that were not able to be sampled.

Of the five major pearl oyster species which are the focus of commercial aquaculture efforts^1,2^, the only species distributed over a natural range comparable to that of *P*. *margaritifera*, is the Akoya complex; *P*. *fucata/martensii/radiata/imbricata*. The Akoya complex is characterised by substantial intra- and interpopulation morphological variability, local geographic isolation of some populations, human introductions, hybridisation and inconsistent taxonomic practice^2,23^. Originally, three distinct species were recognised; *P*. *imbricata* (Röding, 1798; western Atlantic), *P*. *radiata* (Leach, 1814); eastern Indian Ocean and Red Sea) and *P*. *fucata* (Gould, 1850; Indo-Pacific). Japanese populations were recognised as a distinct species (*P*. *martensii*; Dunker, 1872) or subspecies (*P*. *fucata martensii* ^2,39,40^). These classifications were on the basis of questionable morphological characters, and subsequent molecular analyses (see Wada and Tëmkin^2^ and Tëmkin^23^ for summaries), revealed that Australian, south-east Asian and Japanese populations form a monophyletic group that is highly likely to be conspecific. Furthermore, mating experiments have supported the conspecificity of south-east Asian and Japanese populations^41^, and the current consensus is that the Akoya complex may be a cosmopolitan, globally distributed species, possessing substantial intraspecific variation^2^.

Given the similarities in intraspecific morphological variability and the extensive natural distribution of *P*. *margaritifera* when compared with members of the Akoya species complex, in the light of the current study, it is certainly feasible that *P*. *margaritifera* (as it is currently known) might comprise a species complex. Ultimate resolution of its taxonomic identity however, will require a large-scale, systematic, molecular and morphological characterisation of samples collected across the entire natural distribution.

The current study is the first range-wide examination of evolutionary relationships in the black-lip pearl oyster using genome-wide molecular markers. It identifies the presence of discrete ESUs within the species distribution, and presents evidence for the conspecificity of *P*. *mazatlanica* and *P*. *margaritifera*. Collectively, these findings provide early indications that *P*. *margaritifera* may constitute a species complex, and highlight the requirement for further range-wide investigations to fully resolve its taxonomic status. This information is valuable not only for the regional fishery management and aquaculture of *P*. *margaritifera*, as discrete ESUs require independent management^14,22^, but also for a broader understanding of the ecology and evolution of similarly wide-ranging marine invertebrates.

## Methods and Materials

### Specimen collection, tissue sampling and DNA extraction

Specimens between 7–18 cm in dorso-ventral measurement (DVM) were collected from several sites spanning the natural distributions of five Pteriid pearl oyster species (Fig. 1). Black-lip pearl oysters (*Pinctada margaritifera*; n = 69) were sampled from 14 sites, which for the Indian Ocean included five samples from each of two Tanzanian sites; (Mafia Island and Mtwara), and Post Office Island in the Abrolhos Islands group, Western Australia; with four samples from the Persian Gulf (Hendorabi Island, Iran). Five oysters each were also sampled from Western and Central Pacific Ocean populations, including Checheng, Taiwan; Nha Trang, Vietnam; Manado, Indonesia; Kavieng, Papua New Guinea; Gizo Island in the Solomon Islands; Arlington, Sudbury and Tongue Reef systems within the Great Barrier Reef (GBR), Australia; Kadavu, Savusavu, Lau and the Yasawa group, Fiji Islands; and Tongatapu, Tonga; respectively. In the Eastern Pacific, five oysters each were collected from Manihiki Atoll in the Cook Islands, and Arutua, French Polynesia respectively.

Silver-lip pearl oyster (*P*. *maxima*, n = 29) specimens were obtained from Hainan Island, China; Phú Quốc, Vietnam; Broome, Western Australia; Thursday Island in the Torres Straits, Australia; Hervey Bay, eastern Australia and Gizo Island in the Solomon Islands. Five samples were obtained from each of these sites, with the exception of Hervey Bay, where four oysters were collected. *P*. *maxima* was included here as it is the closest known relative of *P*. *margaritifera* ^42^. Panamanian pearl oyster (*P*. *mazatlanica*, n = 10) specimens were also collected from a single site at Guaymas, Mexico, while Akoya pearl oysters (*P*. *fucata martensii*, n = 1 and *P*. *imbricata*, n = 3) were collected from Okinawa, Japan and Port Stephens, Australia; respectively. These additional taxa were selected as they were recovered by Yu and Chu^42^ and Cunha, *et al*.^24^ as separate clades to *P*. *margaritifera*, and consequently included to provide a basis for comparison when using conserved dominant loci in the phylogenomic analysis. Penguin’s winged pearl oyster *Pteria penguin* was selected as the outgroup taxon, with specimens (n = 5) obtained from Savusavu, Fiji Islands.

Proximal mantle and adductor muscle tissues (3 and 6 cm respectively) were removed from each specimen and transferred to tubes containing 20% salt saturated dimethyl sulfoxide (DMSO-salt) preservative^43^. Genomic DNA was extracted using a modified cetyl trimethyl ammonium bromide (CTAB) chloroform/isoamyl alcohol protocol with a warm (30 °C) isopropanol precipitation^44^. To clean up all DNA extractions, a Sephadex G50^45^ spin column protocol was used prior to quantification with a Nanodrop 1000 Spectrophotometer (Thermo Scientific). All samples were subsequently normalised at 100 ng/μL in a 50 μL final volume, and submitted for DArTseq. 1.0 genotyping at Diversity Arrays Technology PL, Canberra, ACT, Australia.

### DArTseq. 1.0 library preparation and sequencing

Diversity Arrays Technology (DArT PL) proprietary genotyping by sequencing (DArTseq) reduced-representation libraries were prepared as described by Kilian, *et al*.^46^ and Sansaloni, *et al*.^47^, with a number of modifications for *P*. *margaritifera*. Briefly, genome complexity reduction was achieved with a double restriction digest, using a *Pst*I and *Sph*I methylation-sensitive restriction enzyme (RE) combination, in a joint digestion-ligation reaction at 37 °C for 2 hr with 150–200 ng gDNA. Because *P*. *margaritifera*, like other bivalve species, is highly polymorphic^12,48^, highly repetitive genomic regions were avoided and low copy regions more efficiently targeted for sequence capture with the use of methylation-sensitive REs^49^.

Custom proprietary barcoded adapters (6–9 bp) were ligated to RE cut-site overhangs as per Kilian, *et al*.^46^, with the adapters designed to modify RE cut sites following ligation, to prevent insert fragment re-digestion. The *Pst*I-compatible (forward) adapter incorporated an Illumina flowcell attachment region, sequencing primer sequence and a varying length barcode region^46,50^. The reverse adapter also contained a flowcell attachment region, and was compatible with the *Sph*I cut-site overhang. Samples were processed in batches of 94, with 15% of all samples in a batch randomly selected for replication, to provide a basis for assessing region recovery and genotyping reproducibility. Target “mixed” fragments^50^, containing both *Sph*I and *Nla*III cut-sites were selectively amplified using custom designed primers for each sample, under the following PCR conditions: initial denaturation at 94 °C for 1 min, then 30 cycles of 94 °C for 20 s, 58 °C for 30 s and 72 °C for 45 s, followed by a final extension step at 72 °C for 7 min. Amplified samples were subsequently cleaned using a GenElute PCR Clean-up Kit (Sigma-Aldrich, cat.# NA1020-1KT), on a TECAN Freedom EVO150 automated liquid handler.

To examine fragment size concordance and digestion efficiency, all samples were visualised on a 0.8% agarose gel stained with EtBr, and quantified using the ImageJ software package^51^. Samples which did not appear to have undergone complete digestion and/or amplification were removed from downstream library preparation. A total of 288 samples were normalised and pooled using an automated liquid handler, at equimolar ratios for sequencing in single lanes on the Illumina HiSeq. 2500 platform. After cluster generation and amplification (HiSeq SR Cluster Kit V4 cBOT, cat.# GD-401-4001), 77 bp single-end sequencing was performed at the DArT PL facility in Canberra, Australia.

### Sequence quality control, marker filtering and genotype calling at DArT PL

Raw reads obtained following sequencing were processed using Illumina CASAVA v.1.8.2 software for initial assessment of read quality, sequence representation and generation of FASTQ files. Filtered FASTQ files were then supplied to the DArT PL proprietary software pipeline DArTtoolbox, which performed further filtering, variant calling and generated final genotypes in sequential primary and secondary workflows. Within DArTtoolbox, the primary workflow first involved the package DArTsoft14 to remove reads with a quality score <25 from further processing, and apply stringent filtering to the barcode region of all sequences to increase confidence in genomic region recovery. Individual samples were then de-multiplexed by barcode, and subsequently aligned and matched to catalogued sequences in both NCBI GenBank and DArTdb custom databases to check for viral and bacterial contamination, with any matches removed from further processing.

The secondary workflow employed the DArTsoft14 and KD Compute packages along with the DArTdb database, to identify polymorphisms by aligning identical reads to create clusters across all individuals sequenced. These clusters were then catalogued in DArTdb, and matched against each other to create reduced-representation loci (RRL), based on their degree of similarity and size. SNP and reference allele loci were identified within clusters and assigned DArT scores (“0” = reference allele homozygote, “1” = SNP allele homozygote and “2” = heterozygote), based on their frequency of occurrence. To ensure robust variant calling, all monomorphic clusters were removed, SNP loci had to be present in both allelic states (homozygous and heterozygous) and a genetic similarity matrix produced using the first 10,000 SNPs called was used to assess technical replication error^52^. Gene duplications were eliminated by excluding clusters containing tri-allelic or aberrant SNPs and overrepresented sequences.

Presence-absence variant (PAV) markers (termed SilicoDArT loci) were also identified using restriction site-associated (RAD) fragments recovered in the sequence data. SilicoDArTs were scored in a binary fashion, with “1” = RAD fragment presence, “0” = RAD fragment absence and “-“ = insufficient counts re-classified as “1”; indicating a hemizygote state. DArTseq PAV markers can be considered to be genome-wide “dominant” markers^53–55^, and were called based on a minimum reproducibility of 95%. Once SNP and PAV markers had been confidently identified, each locus was assessed in the KD Compute package for homozygote and heterozygote call rate, frequency, polymorphic information content (PIC), average SNP count, read depth and repeatability, before final genotype scores were supplied by DArT PL.

Following the receipt of genotype data from DArT PL, the SNP dataset was initially filtered using a custom Python script (https://github.com/esteinig/dartQC). This script retained only a single informative SNP (determined by call rate) at each genomic locus, and then filtered all SNPs at a Minor Allele Frequency (MAF) of 2%. Final filtering of the SNP dataset was by call rate (>70%), read depth (>4) and reproducibility (>95%). A total of 11 individuals across 3 taxa; (a single *P*. *margaritifera* individual sampled from Iran, along with all *Pt*. *penguin*, *P*. *imbricata* and *P*. *fucata martensii* specimens), were excluded from the final dataset due to poor call rates (<1%). These taxa were the most divergent compared to all others considered for analysis, and likely experienced poor genotyping coverage due to severe allelic dropout. Consequently, all analyses using the SNP dataset were assigned *P*. *maxima* as the outgroup. PAV markers were filtered manually, first to retain the most informative marker at each genomic locus, and then in the order of call rate (100%), MAF (>9.6%), reproducibility (>98%) and read depth (>113).

### Assessment of differentiation between population groups and species

Nei’s (1973) minimum genetic distances (*D*_m_) between populations were computed in Genetix v.4.05.2 with 1,000 permutations^56^ for the SNP dataset, while for the PAV dataset, Nei’s unbiased (1972) genetic distances (*D*) were calculated after Lynch and Milligan^57^, using the AFLP-SURV v.1.0 package and 10,000 permutations^58,59^. Estimates were calculated for both the SNP and PAV datasets, to permit comparison between the two marker types. Computations using the PAV dataset had to be limited to 5,000 loci, as this was the maximum number of sites able to be handled by the software. Random sub-sampling of different sets of 5,000 loci from the larger dataset, and recalculation of genetic distances ensured estimates remained unaffected.

### Phylogenomic reconstruction

Population and species-level relationships were reconstructed using neighbour-joining (NJ), maximum-likelihood (ML) and Bayesian inference approaches (see Supplementary Figure 1 for a summary). The NJ tree was constructed in the MEGA6 software package^60^. A matrix of Nei’s (1972) genetic distances calculated in Genetix v.4.05^56^ was used as input, and the consensus tree generated over 1000 bootstraps using the SNP dataset. The outgroup taxon selected was *P*. *maxima*, as the more distant outgroup of *Pt*. *penguin* was highly divergent from *P*. *margaritifera*, and as a result received very low genotyping coverage in the SNP dataset (>99% missing data, see results).

Additionally, the SNPhylo^61^ and RAxML v8.2^62^ packages were both used to perform ML analyses on the SNP dataset, with RAxML only for the PAV dataset. As the SNP dataset had already been filtered, for SNPhylo computations, the -r flag was selected which bypassed filtering options for low SNP sample coverage and missing data, and the -l flag set at 1.0 to ignore filtering for linkage disequilibrium (LD) for the final analysis. Preliminary runs were performed both with and without LD pruning to ascertain if this effected changes in tree topology, and no rearrangement of higher-level groups was observed. The transition/transversion ratio was set at 2.0 (−T flag), empirical base frequencies used (−F flag), and constant rate variation set among sites (−R flag). All other options remained at their default settings. SNPhylo first performs a multiple sequence alignment by MUltiple Sequence Comparison by Log-Expectation (MUSCLE; ref.^63^), and generates a ML tree using the DNAML program in the PHYLIP package^64^, with bootstrap support provided by the *R* package *phangorn* ^65^. The final tree was generated following 100,000 bootstraps, with *P*. *maxima* used as the outgroup taxon. RAxML analyses used the site-specific heterogeneity models^66^ ASC_GTRGAMMA[X] (for SNPs) and ASC_BINCAT[X] (for PAVs), with the following options selected: ascertainment bias correction set to ‘Lewis’, the rapid bootstrap algorithm with ‘autoMRE’ option enabled and retention of the best ML tree^62,67,68^. All RAxML reconstructions showed nearly identical topologies for the SNP dataset, and supported the relationships recovered by the Bayesian reconstruction for the PAV dataset.

Bayesian inference of phylogenetic relationships was carried out using only the PAV dataset, with the MrBayes v3.2 package^69–71^ and parameter settings were adapted after Koopman, *et al*.^72^. The analysis incorporated two runs of 60,000,000 generations each, with each run comprising 4 independent chains. A temperature of 0.10 was set for the heated chains, with a sampling frequency of 1000 and burn-in fraction of 20%. The burn-in threshold was selected on the basis that both independent runs had achieved convergence (*i*.*e*. stable log likelihood values reached for all sampled trees, gauged by the average standard deviation of split frequencies). Convergence was also independently assessed using Tracer v.1.6^73^. The Dirichlet prior for state frequencies was set at (90, 10), matching the frequencies of “0” and “1” PAV scores present in the dataset. The outgroup taxon set incorporated all individuals of *Pteria penguin*, together with the additional taxa *P*. *imbricata* and *P*. *fucata martensii*. Inclusion of these samples became possible as the PAV dataset was more informative compared to the SNP dataset, for these more evolutionarily distant specimens.

The final trees for the Bayesian analysis were generated by selecting only those post-burn in trees found with the highest individual and cumulative posterior probabilities (p = 0.000 and P < 0.015 respectively), during Markov Chain Monte Carlo (MCMC) computations. A consensus tree was then constructed from this final credible set of trees using a 50% majority consensus rule in the Dendroscope 3.5.7 package^74^. All phylograms were visualised, inspected and edited in FigTree v.1.4.2^75^.

### Data availability

Genotypic data have been provided as supplementary datasets. Incorporated in text.

## Electronic supplementary material

Supplementary Figure 1

Supplementary Dataset 1

Supplementary Dataset 2
