# Supplementary material for: Genome-wide comparisons reveal evidence for a species complex in the black-lip pearl oyster Pinctada margaritifera (Bivalvia: Pteriidae)
Source: Sci Rep. 2018 Jan 9;8:191. doi: 10.1038/s41598-017-18602-5 (PMC5760631; doi:10.1038/s41598-017-18602-5)
Supplement: Supplementary file 1 — Supplementary Figure 1 [file 41598_2017_18602_MOESM1_ESM.pdf]

Supplementary Figure 1

Supplementary information for:

Genome-wide comparisons reveal evidence for a species complex in the black-lip pearl oyster *Pinctada margaritifera* (Bivalvia: Pteriidae)

Monal M. Lal, Paul C. Southgate, Dean R. Jerry and Kyall R. Zenger

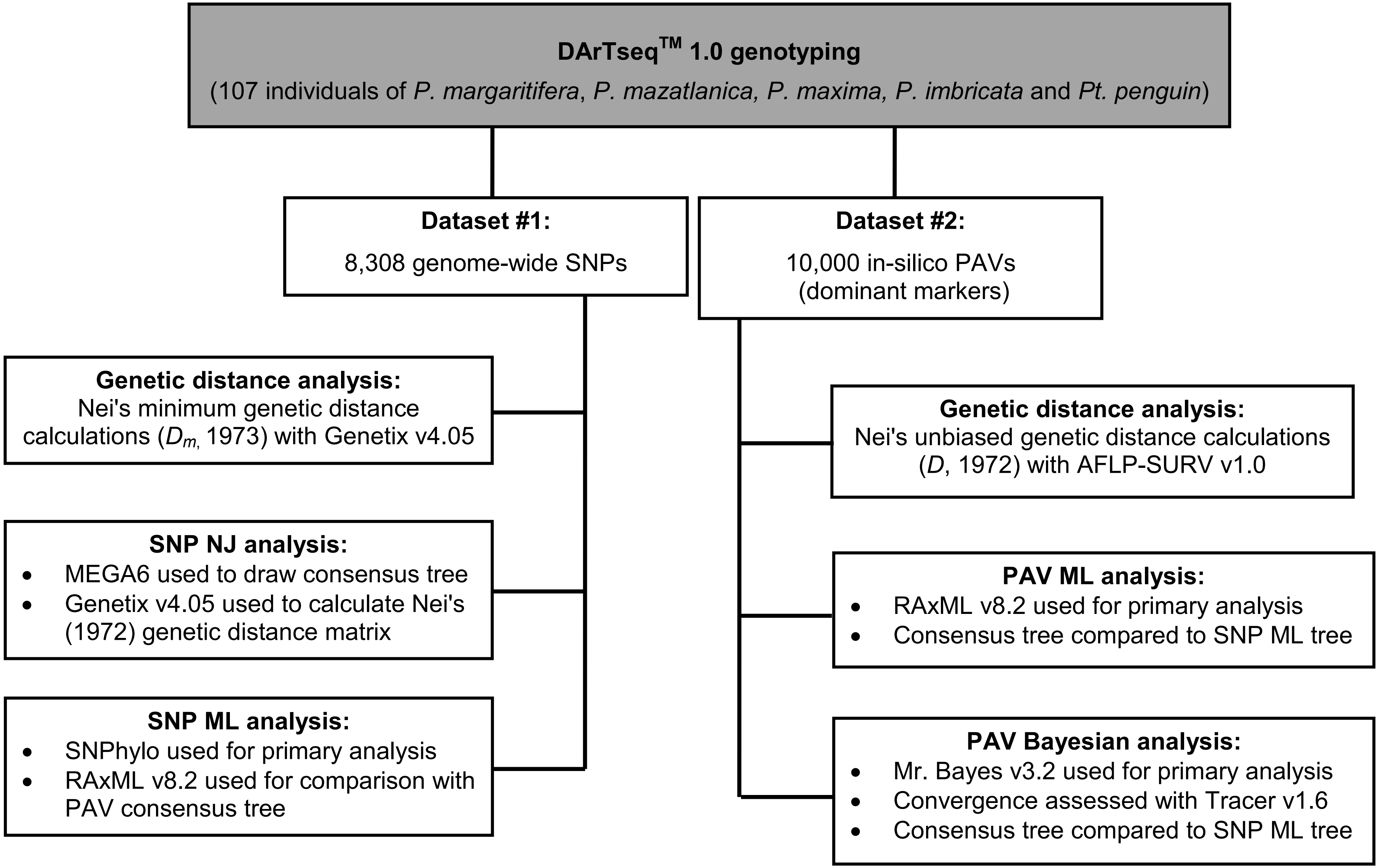
DArTseq™ 1.0 genotyping
(107 individuals of P. margaritifera, P. mazatlanica, P. maxima, P. imbricata and Pt. penguin)
Dataset #1:
8,308 genome-wide SNPs
Dataset #2:
10,000 in-silico PAVs (dominant markers)
Genetic distance analysis:
Nei's minimum genetic distance calculations (D_m, 1973) with Genetix v4.05
SNP NJ analysis:
• MEGA6 used to draw consensus tree
• Genetix v4.05 used to calculate Nei's (1972) genetic distance matrix
SNP ML analysis:
• SNPhylo used for primary analysis
• RAxML v8.2 used for comparison with PAV consensus tree
Genetic distance analysis:
Nei's unbiased genetic distance calculations (D, 1972) with AFLP-SURV v1.0
PAV ML analysis:
• RAxML v8.2 used for primary analysis
• Consensus tree compared to SNP ML tree
PAV Bayesian analysis:
• Mr. Bayes v3.2 used for primary analysis
• Convergence assessed with Tracer v1.6
• Consensus tree compared to SNP ML tree
